# Variation in fecal hemoglobin concentrations: Cross-sectional analysis of a screening trial and a screening program in Sweden

**DOI:** 10.1177/09691413251369323

**Published:** 2025-08-21

**Authors:** Masau Sekiguchi, Christian Löwbeer, Robert Steele, Johannes Blom, Anna Forsberg, Marcus Westerberg

**Affiliations:** 1 Endoscopy Division/Cancer Screening Center, National Cancer Center Hospital, Tokyo, Japan; 2 Division of Screening Technology, National Cancer Center Institute for Cancer Control, Tokyo, Japan; 3 Department of Laboratory Medicine, Division of Clinical Chemistry, and Department of Clinical Science and Education, 27106 Södersjukhuset, Karolinska Institutet, Stockholm, Sweden; 4 Department of Clinical Chemistry, SYNLAB Sverige, Täby, Sweden; 5 Department of Surgery, Population Health and Genomics, School of Medicine, University of Dundee, Ninewells Hospital, Dundee, Scotland, UK; 6 Department of Surgery, 27106 Södersjukhuset, Karolinska Institutet, Stockholm, Sweden; 7 Department of Clinical Science and Education, Karolinska Institutet, Stockholm, Sweden; 8 Division of Clinical Epidemiology, Department of Medicine K2, 27106 Solna, Karolinska Institutet, Stockholm, Sweden; 9 Department of Surgical Sciences, 8097 Uppsala University, Uppsala, Sweden

**Keywords:** Colorectal cancer, fecal immunochemical test, screening

## Abstract

**Objectives:**

To assess variation in fecal hemoglobin concentration according to year and season of fecal immunochemical test screening in Sweden, the detection rate of advanced neoplasia, and factors that could influence fecal immunochemical test positivity including sex, age, comorbidity, and laboratory testing quality.

**Methods:**

We performed a cross-sectional analysis of participants in the fecal immunochemical test arm of the randomized controlled trial SCREESCO between March 2014 and December 2019 and of participants in the screening program of Stockholm–Gotland, Sweden, who underwent a one-sample fecal immunochemical test between October 2015 and October 2024.

**Results:**

A total of 33,232 individuals from SCREESCO and 315,664 individuals from the Stockholm–Gotland screening program were included. Fecal immunochemical test hemoglobin concentrations were generally higher in the winter but this varied over calendar years. In SCREESCO, the median fecal immunochemical test concentration was 0.0 μg hemoglobin/g feces in December 2015, 1.0 μg hemoglobin/g feces in June and 5.0 μg hemoglobin/g feces in December 2016, and 0.0 μg hemoglobin/g feces in June 2017. This was paralleled by a similar variation in the Stockholm–Gotland screening program. In months with higher fecal immunochemical test positivity in SCREESCO, there was a higher number of colonoscopies and a lower rate of advanced neoplasia detected. Male sex, higher age, and higher comorbidity were also associated with higher fecal immunochemical test positivity.

**Conclusions:**

The variation in the number of colonoscopies and detection rate of advanced neoplasia paralleled the seasonal variation in fecal immunochemical test and warrants further studies on seasonal variation of fecal immunochemical test to optimize fecal immunochemical test-based colorectal cancer screening.

## Introduction

A fecal immunochemical test (FIT) is widely used for colorectal cancer (CRC) screening.^1^ A positive FIT is usually followed by a colonoscopy that detects and, when appropriate, removes CRC and pre-cancerous lesions with the potential to reduce CRC incidence and mortality.^2,3^ However, the instability of hemoglobin (Hb) presents challenges in ensuring the consistent performance of FIT.^2^

Factors influencing FIT performance and outcomes have been explored in previous studies.^4–20^ Several participant-related factors, including age and sex, are known to be associated with test outcomes.^4–6^ The impact of ambient temperature and seasonal variations on FIT Hb concentration, positivity, and sensitivity has been discrepant across countries, potentially due to geographical differences.^5–15^ Given that factors influencing FIT performance have been sparsely examined in northern Europe, investigating the variation in FIT Hb concentration, giving consideration to various participant-, environmental-, and laboratory-related factors in this region, would be informative.

In this study, we aimed to assess variation in fecal Hb concentration according to calendar year and season in individuals participating in FIT screening in SCREESCO (SCREEning of Swedish Colons) and in the Stockholm–Gotland screening program. We also aimed to assess the detection rate of advanced neoplasia according to calendar year and season in individuals participating in FIT screening in SCREESCO, and factors that potentially influence FIT positivity.

## Materials and methods

### Study population

SCREESCO is a randomized clinical trial (RCT) conducted in 18 out of 21 regions of Sweden that includes a FIT screening arm. Two of the regions not included in SCREESCO, Stockholm and Gotland, have had population-based biennial guaiac fecal occult blood test (gFOBT) screening in the 60–69-year-old population since 2008 and with one-sample FIT since October 2015, with a cut-off level of 40 µg Hb/g feces for a positive test in women and 80 µg Hb/g in men.^21,22^

We performed a cross-sectional analysis of participants in the FIT × 2 arm of SCREESCO,^23, 24^ who underwent a screening FIT between March 2014 and December 2019, and of participants in the regional population-based screening program of Stockholm–Gotland, Sweden, who underwent a screening FIT between October 2015 and October 2024.^21^ Participants in the FIT arm of SCREESCO were included in the present study if they participated in the FIT screening and returned at least two non-faulty tests within the first screening round in which they participated. All participants in the Stockholm–Gotland screening program were included if they underwent at least one non-faulty FIT.

### About SCREESCO

In SCREESCO, 60-year-old men and women (born 1954–1958) residing in any of the 18 regions of Sweden where screening for CRC had not previously being offered, and without a previous diagnosis of colorectal or anal cancer, or having participated in the The Northern-European Initiative on Colorectal Cancer (NordICC) trial,^25^ were randomized to one of the three arms: once-only primary colonoscopy, invitations to two rounds of high-sensitive FIT using a kit for stool samples 2 years apart (FIT × 2 arm), or no intervention (control arm) as previously described.^23,24^

Individuals assigned to the FIT arm were sent kits for fecal samples. A quantitative FIT using two fecal samples, with separate kits for each fecal sample, was offered in each screening round. The invitees were instructed to take samples from two different bowel movements, preferably not on the same day. One central laboratory performed all FIT analyses using the same method, staff, and a single OC-Sensor Diana automated analyzer. Individuals were invited to a colonoscopy if at least one of the two stool samples had a fecal Hb concentration of at least 10 μg Hb/g feces. If both stool samples were faulty or if one was faulty and the other had a Hb concentration below 10 μg Hb/g feces, then a new a kit for stool samples was sent. Individuals with a fecal Hb concentration below 10 μg/g in both stool samples were considered test negative and not offered a follow-up colonoscopy. All individuals in the FIT arm, except those requiring colonoscopy surveillance after adenoma removal or after a CRC diagnosis, were offered a repeat FIT after 2 years, irrespective of participation in the first FIT screening round or whether the result was negative or positive.

All analyses of the FITs in SCREESCO were carried out by four trained and certified laboratory staff, working according to standard operating procedures. The laboratory had a total quality management system and was accredited to ISO 15189:2012 standards by the Swedish Board for Accreditation and Conformity Assessment, Swedac (Borås, Sweden). The FIT method was calibrated once per month with the calibrators provided (OC-Calibrator 1). Each analytical run was preceded by analyses of the internal quality control materials: the low target (range of targets 6–10 μg Hb/g feces, range of standard deviations 0.5–0.8 μg Hb/g feces), and high target (range of targets 78–94.4 μg Hb/g feces, range of standard deviations 3.1–3.8 μg Hb/g feces) f-Hb OC-Control (Eiken Chemical Co., Ltd, Tokyo, Japan). These quality control materials were performed every 200 tests analyzed.

### About the Stockholm screening program

The screening program of Stockholm–Gotland has an uptake of around 25% of the Swedish population, and by 2014 all aged 60–69 had been invited.^26^ The invitations were sent by mail biennially for 10 years, that is, five screening rounds. The invitations included information on CRC screening, a test kit with three gFOBTs (Hemoccult-test, Beckman Coulter, IN, USA), instructions on how to take the test, and a prepaid return envelope to the laboratory for analysis. Participants with negative test results were informed by letter and reinvited in 2 years; the individuals with positive tests were electronically referred to a follow-up diagnostic colonoscopy within 2 weeks. Participants with faulty tests were sent new test kits and non-responders were sent a reminder after 8 weeks.

On October 1st, 2015, the program changed to one-sample FIT f-Hb OC-Sensor Diana (Eiken Chemical Co., Ltd, Tokyo, Japan) with a cut-off level of 40 µg Hb/g feces for a positive test in women and 80 µg Hb/g in men.^21^ The analyses of the FITs were performed in the same laboratory as the SCREESCO FITs until 2019. Karolinska University Laboratory completely took over the Stockholm–Gotland FIT screening on January 1st, 2019 and used OC-Sensor Pledia until September 2023, when they switched to the FOBGold FIT (SYSMEX) method.

### Ethics

The Stockholm Ethics Committee approved the SCREESCO study (2012/2058-31/3) and the review of medical charts (2015/1958-2). Individuals performed informed consent by returning an FIT. The Swedish Ethical Review Authority waived the need for informed consent for accessing pseudonymized register-based data on individuals in SCREESCO (2022/01946-02 and 2022/06863-2).^27^ The evaluation of the FIT used in the Stockholm–Gotland screening program was approved by the Swedish Ethical Review Authority (2019-04850 and 2023-00400-02).

### Data

Data from the Cancer Register, the Patient Register, and the Prescribed Drug Register at the National Board of Health and Welfare, and the Total Population Register at Statistics Sweden, were linked to the SCREESCO database and pseudonymized. Age, sex, region of residence, dates and values of all FITs, and findings of advanced neoplasia (comprising CRC and advanced adenomas—defined as adenomas with a diameter of ≥10 mm, high-grade dysplasia, or a prominent villous component) at screening colonoscopies were extracted from the SCREESCO database.^28^ Internal quality control FITs were extracted from the laboratory (Synlab, Stockholm, Sweden) and were standardized by subtracting the target level from the measured level and dividing by the standard deviation. To assess potential issues in FIT quality at the date of a FIT performed in SCREESCO, we assigned to each FIT the closest (in time) quality control FIT value and expected standard deviation, and computed the standardized laboratory FIT control difference: (FIT value − control FIT value)/expected standard deviation.

The Charlson comorbidity index (CCI) was calculated based on the Patient Register in the 10 years preceding the date of FIT.^29^ A drug comorbidity index (DCI) was calculated based on filled drug prescriptions registered up to 1 year before date of the FIT in the Prescribed Drug Register.^30,31^ The DCI is a weighted sum, similar to the CCI, based on the Anatomical Therapeutic Chemical classification system codes of filled drug prescriptions and has been shown to discriminate risk of death better than the CCI in both men and women.

Monthly aggregated data were extracted from the Stockholm–Gotland screening program.

### Statistical analysis

The larger of the two FITs in SCREESCO were used throughout the analysis. The monthly number of FIT participants and tests in SCREESCO and the Stockholm screening program was extracted along with the median and 90% quantile (*Q*_90_) of the FIT Hb concentrations.

The proportion of positive FIT, proportion of colonoscopies, and proportion of colonoscopies positive for advanced adenoma or CRC in SCREESCO were extracted. The cut-off for a positive FIT was 10 μg Hb/g feces in SCREESCO but in complementary analyses we additionally considered 40 and 80 μg Hb/g feces. Analyses were performed according to calendar time (monthly) and season (monthly).

A multivariable generalized additive logistic regression model was used to assess the association between FIT positivity and age at FIT, sex, calendar time of FIT, time from sampling to FIT analysis, season of FIT, internal FIT control (low and high), the CCI, and the DCI. All variables except for age, sex, CCI, and DCI were modeled using thin plate regression splines. We computed 95% confidence intervals (CIs) for the odds ratios (ORs) using bootstrap (*N* = 500 replications) where resampling was performed on the level of individuals.

The analyses were performed using R (4.1.3).

## Results

### Baseline characteristics

In the SCREESCO study, 60,300 were randomized to the FIT arm, and 33,232 (55%) out of 60,137 invited returned at least two non-faulty FIT in the first screening round and were included in this study. Median FIT concentration was 1.2 μg Hb/g feces (*Q*_90_: 8.4 μg Hb/g feces) and most FITs (*n* = 60,697; 91.3%) were negative (<10 μg Hb/g feces) (Table 1). Most individuals (*n* = 28,025, 84%) completed FIT between December 2014 and June 2018. The laboratory FIT control difference (low) varied somewhat according to calendar time, for example, from −0.6 (interquartile range (IQR): −1.0 to 0.0) in 2016 and 0.7 (IQR: −0.6 to 1.5) in 2017, and the median time from sampling to FIT analysis was 3 days (2–4 days). For participants that underwent a colonoscopy the median time from a positive FIT to colonoscopy was 43 days (IQR: 34–60 days).

**Table 1.** Baseline characteristics of screening participants and fecal immunochemical tests in SCREESCO.

	Year of FIT							
	2014–2015		2016		2017		2018–2019	
*N* individuals	14,776	(100)	11,313	(100)	4730	(100)	2413	(100)
*N* FIT	29,550	(100)	22,608	(100)	9480	(100)	4826	(100)
Sex								
Male	13,359	(45.2)	10,425	(46.1)	4612	(48.6)	2480	(51.4)
Female	16,191	(54.8)	12,183	(53.9)	4868	(51.4)	2346	(48.6)
Age at FIT (y)								
Median (IQR)	60.5 (60.3–60.7)		60.6 (60.4–60.8)		60.5 (60.3–62.6)		62.6 (62.4–62.8)	
59	2861	(9.7)	0	(0.0)	2	(0.0)	0	(0.0)
60	25,941	(87.8)	20,397	(90.2)	5807	(61.3)	0	(0.0)
61	748	(2.5)	1812	(8)	304	(3.2)	8	(0.2)
62–65	0	(0.0)	399	(1.8)	3367	(35.5)	4818	(99.8)
Season of FIT								
Winter (Dec–Feb)	9849	(33.3)	5077	(22.5)	4589	(48.4)	1414	(29.3)
Spring (Mar–May)	7648	(25.9)	5787	(25.6)	3141	(33.1)	1408	(29.2)
Summer (Jun–Aug)	3673	(12.4)	2492	(11.0)	760	(8.0)	704	(14.6)
Fall (Sept–Nov)	8380	(28.4)	9252	(40.9)	990	(10.4)	1300	(26.9)
FIT concentration (μg Hb/g feces)								
Median (IQR)	0.4 (0.0–1.8)		2.4 (0.4–6.0)		2.8 (0.6–5.0)		0.6 (0.0–3.0)	
0–9.9	27,379	(92.7)	20,523	(90.8)	8417	(88.8)	4378	(90.7)
10–19.9	765	(2.6)	888	(3.9)	470	(5)	173	(3.6)
20–39.9	536	(1.8)	512	(2.3)	209	(2.2)	102	(2.1)
40–79.9	360	(1.2)	258	(1.1)	155	(1.6)	64	(1.3)
80+	510	(1.7)	427	(1.9)	229	(2.4)	109	(2.3)
Laboratory FIT control difference (low)								
Median (IQR)	−0.3 (−0.8 to 0.2)		−0.6 (−1 to 0)		0.7 (−0.6 to 1.5)		0.1 (−0.6 to 0.9)	
<−2	186	(0.6)	1082	(4.8)	354	(3.7)	50	(1.0)
−2 to −1	3677	(12.4)	4489	(19.9)	1100	(11.6)	582	(12.1)
−1 to 0	13,505	(45.7)	10,903	(48.2)	1708	(18.0)	1416	(29.3)
0–1	10,034	(34.0)	3557	(15.7)	2431	(25.6)	1726	(35.8)
1–2	974	(3.3)	1353	(6.0)	2301	(24.3)	634	(13.1)
>2	1174	(4.0)	1224	(5.4)	1586	(16.7)	418	(8.7)
Laboratory FIT control difference (high)								
Median (IQR)	−0.4 (−1.0 to 0.4)		0.2 (−0.4 to 1.0)		−0.3 (−0.9 to 0.5)		0.5 (−0.2 to 1.2)	
<−2	338	(1.1)	528	(2.3)	130	(1.4)	58	(1.2)
−2 to −1	7131	(24.1)	854	(3.8)	1933	(20.4)	266	(5.5)
−1 to 0	11,246	(38.1)	7978	(35.3)	3835	(40.5)	1174	(24.3)
0–1	8651	(29.3)	7728	(34.2)	2122	(22.4)	1830	(37.9)
1–2	2184	(7.4)	4462	(19.7)	1108	(11.7)	1276	(26.4)
>2	0	(0.0)	1058	(4.7)	352	(3.7)	222	(4.6)
Time from sampling to FIT analysis (days)								
Median (IQR)	3 (2–4)		3 (2–4)		3 (2–4)		3 (2–4)	
0–1	5221	(17.7)	4391	(19.4)	1741	(18.4)	865	(17.9)
2–7	22,509	(76.2)	17,795	(78.7)	7554	(79.7)	3855	(79.9)
≥8	1820	(6.2)	422	(1.9)	185	(2.0)	106	(2.2)
Charlson comorbidity index								
0	23,623	(79.9)	18,007	(79.6)	7350	(77.5)	3646	(75.5)
1	2907	(9.8)	2159	(9.5)	1062	(11.2)	536	(11.1)
2	2218	(7.5)	1753	(7.8)	761	(8.0)	412	(8.5)
≥3	802	(2.7)	689	(3)	307	(3.2)	232	(4.8)
Drug comorbidity index								
≤Q1	4139	(14)	2957	(13.1)	1170	(12.3)	517	(10.7)
Q1–Q2	11,080	(37.5)	8426	(37.3)	3310	(34.9)	1628	(33.7)
Q2–Q3	7438	(25.2)	5661	(25.0)	2352	(24.8)	1170	(24.2)
>Q3	6893	(23.3)	5564	(24.6)	2648	(27.9)	1511	(31.3)

FIT: fecal immunochemical test; IQR: interquartile range; Q1: first quartile; Q2: median; Q3: third quartile.

There were 315,664 unique individuals that completed FIT in the Stockholm–Gotland screening program during the study period, and the median FIT concentration was 0.0 μg Hb/g feces (*Q*_90_: 7.0 μg Hb/g feces). The monthly number of individuals undergoing FIT varied between 720 and 16,668.

### Variation according to calendar year and month

**Figure 1.**
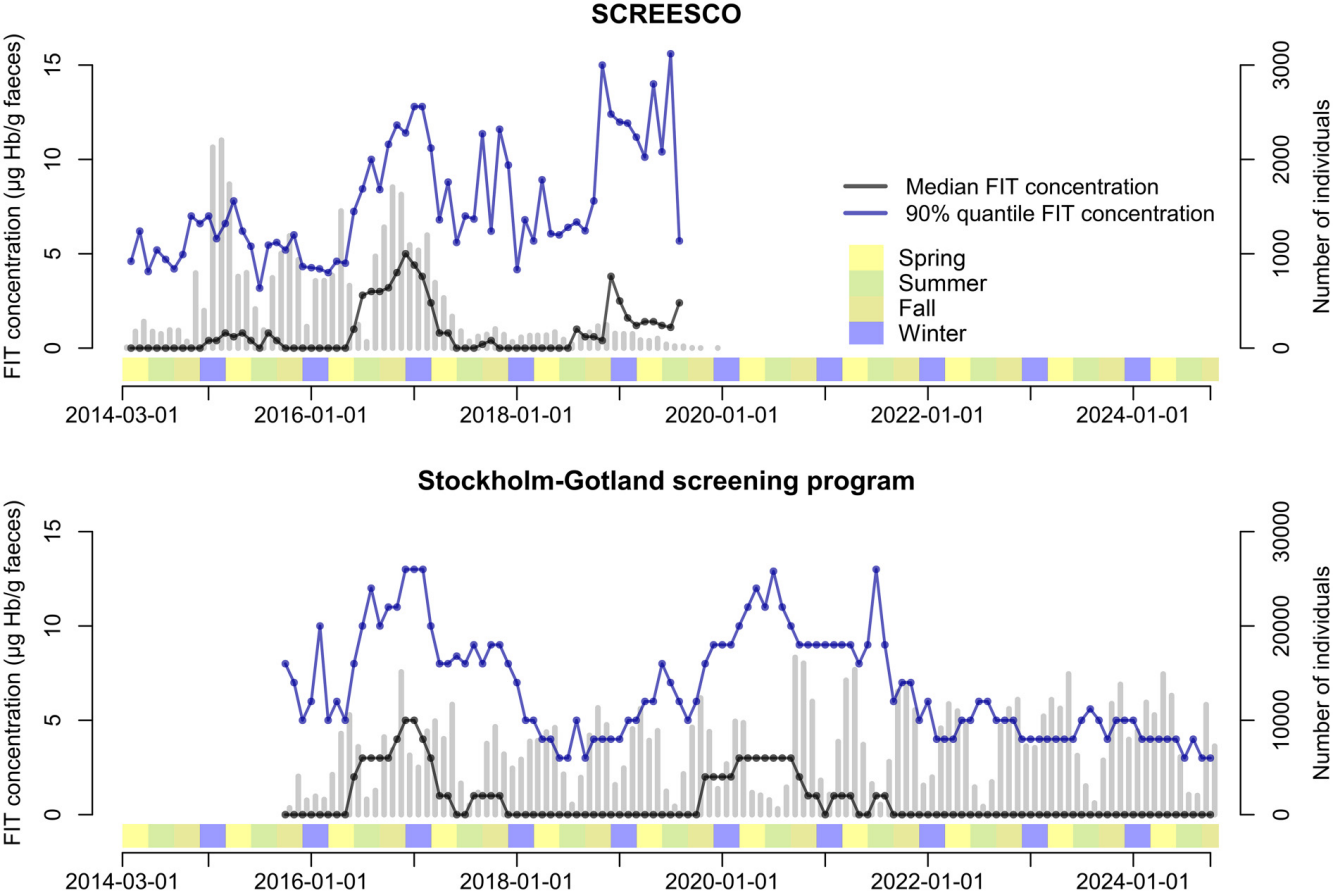
Fecal concentration according to calendar time in SCREESCO and in the Stockholm–Gotland screening program. Gray vertical bars indicate the number of individuals that performed a FIT. Seasons: spring (March–May), summer (June–August), fall (September–November), and winter (December–February). SCREESCO: SCREEning of Swedish Colons; FIT: fecal immunochemical test.

The FIT concentration in SCREESCO varied according to calendar time, with a peak in the winter of 2016–2017 and an increase in FIT concentration from second half of 2018 (Table 1, Figure 1). For example, the median FIT concentration was 0.0 μg Hb/g feces in December 2015 (*Q*_90_ 4.3 μg Hb/g feces), 1.0 μg Hb/g feces in June 2016 (*Q*_90_ 7.2 μg Hb/g feces), 5.0 μg Hb/g feces in December 2016 (*Q*_90_ 11.5 μg Hb/g feces), and 0.0 μg Hb/g feces in June 2017 (*Q*_90_ 5.6 μg Hb/g feces). A parallel variation in FIT concentration was observed in the Stockholm–Gotland screening program, for example, the median FIT concentration was 0.0 μg Hb/g feces in December 2015 (*Q*_90_ 5.0 μg Hb/g feces) and 5.0 μg Hb/g feces in December 2016 (*Q*_90_ 13.0 μg Hb/g feces) (Figure 1). There was also a similar increase in Stockholm–Gotland from the second half of 2018 that peaked around 2020, and in January 2021 the median FIT concentration returned to 0 μg Hb/g feces (*Q*_90_ 6.0 μg Hb/g feces).

### FIT positivity, proportion of colonoscopies, and detection of advanced neoplasia in SCREESCO

**Figure 2.**
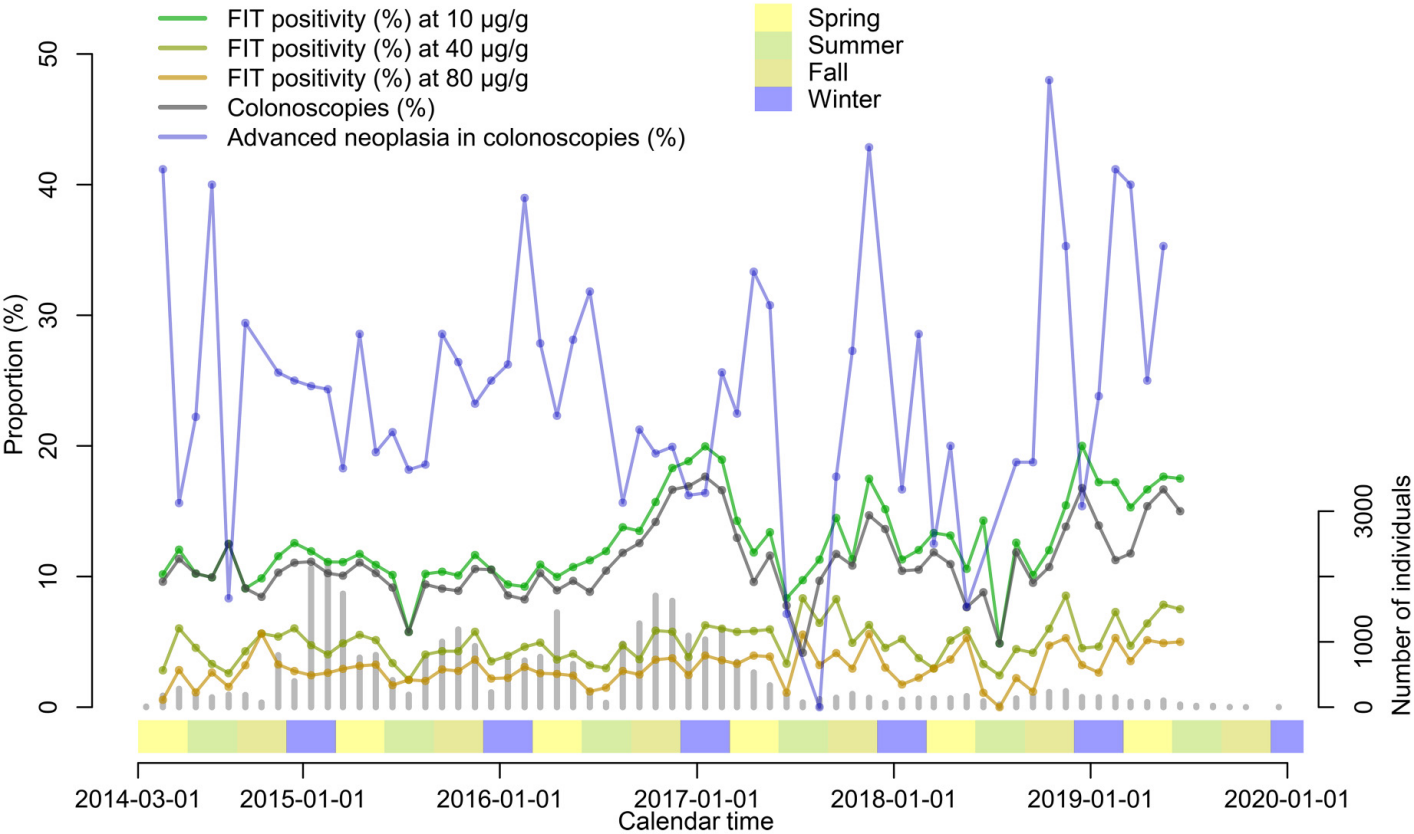
FIT positivity (any of the two tests ≥10 μg Hb/g feces), proportion of colonoscopies in individuals with positive FIT and proportion of advanced neoplasia detected in colonoscopies, in SCREESCO. Gray vertical bars indicate the number of individuals that performed a FIT. Seasons: spring (March–May), summer (June–August), fall (September–November), and winter (December–February). FIT: fecal immunochemical test; Hb: hemoglobin; SCREESCO: SCREEning of Swedish Colons.

Of the 4275 SCREESCO participants with a positive FIT (greater of the two tests), 3840 (90%) underwent a colonoscopy and out of these 882 (23%) had advanced neoplasia detected at colonoscopy. The FIT positivity and proportion of colonoscopies among FIT participants varied in parallel over calendar time and was highest in the winter of 2016–2017 while the proportion of advanced neoplasia was somewhat lower in the winter of 2016–2017 compared to earlier (Figure 2). For example, the proportion of advanced neoplasia among those that underwent a colonoscopy was lower (16%) in December 2016, when the FIT positivity was 19%, compared to December 2014 (25%) and December 2015 (25%), when the FIT positivity was 14% and 11%, respectively.

### Factors associated with FIT positivity

**Figure 3.**
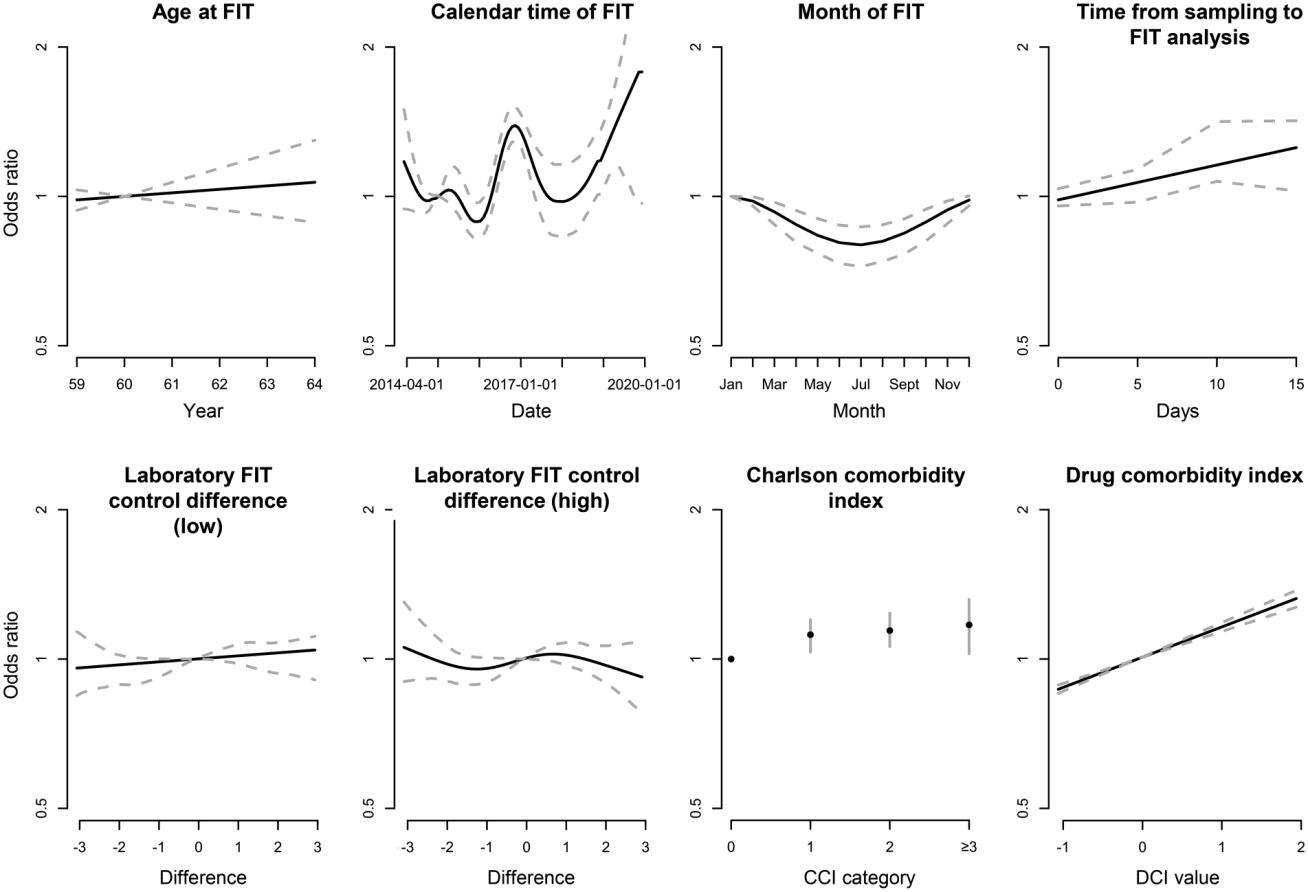
Odds ratios for FIT positivity (any of the two tests ≥10 μg Hb/g feces) in SCREESCO using a multivariable model. Dotted lines are for continuous variables and vertical bars (for Charlson comorbidity index) represent 95% confidence intervals. FIT: fecal immunochemical test; Hb: hemoglobin; SCREESCO: SCREEning of Swedish Colons.

In SCREESCO, women had a lower risk of a positive FIT than men (OR: 0.77; 95% CI: 0.72–0.82). Higher CCI (OR: 1 vs 0: 1.18; 95% CI: 1.05–1.30) and DCI (OR: 1.22; 95% CI: 1.19–1.26) were associated with a higher risk of positive FIT while a higher age was somewhat associated with a higher positivity (OR: 1.02; 95% CI: 0.96–0.1.10) (Figure 3, Supplemental Table 1). Notably, the variation according to calendar time remained when accounting for the other factors, including a lower FIT positivity during the summer compared to the winter, and FIT positivity higher when time from sampling to FIT analysis was around 10 days compared to shorter times (OR: 10 days vs 1 day: 1.23; 95% CI: 1.11–1.65).

Results were largely similar for cut-offs 40 and 80 μg Hb/g feces for a positive FIT, but the variation according to calendar time became less clear with increasing cut-off (Supplemental Figures 1 and 2 and Table 1).

## Discussion

### Summary of findings

There was a substantial variation in FIT Hb concentration in Sweden, in both the screening trial SCREESCO and the regional screening program in Stockholm–Gotland. The seasonal variation persisted even after accounting for age, comorbidity, and laboratory control deviations in the SCREESCO screening trial. Higher fecal Hb concentrations were observed during the winter of 2016–2017, which were also mirrored in the Stockholm–Gotland screening program, followed by another peak around 2020. In months with higher FIT positivity in SCREESCO, there was an increase in the number of screening colonoscopies and a lower detection rate of advanced neoplasia detected in those colonoscopies. Male sex, higher age, and higher comorbidity were also associated with higher FIT positivity in SCREESCO.

### Strengths and limitations

This study has several strengths. SCREESCO is a large RCT covering most of Sweden in a screening-naïve population that has a unique FIT screening design based on two stool samples, and we additionally had access to FIT levels in participants of the Stockholm–Gotland screening program. The participation in the FIT arm of SCREESCO was high for both men and women, and the adherence to colonoscopy following a positive FIT was also high for both sexes (91%) compared to between 64.1% and 92.2% in European FIT screening programs.^23,32^ As only experienced endoscopists who had performed over 100 colonoscopies annually and over 1000 procedures in total performed colonoscopies in SCREESCO, lesion detectability was mostly acceptable although with room for improvement, and comparable with the NordICC study.^25,33^ Nevertheless, some endoscopists did not meet the contemporary international requirement for adenoma detection rate.^34^

The participation rate in the Stockholm–Gotland screening program increased by approximately 10% with a change of test in 2015,^21^ and was on average 63.3% during a maximum of 14 years of follow-up, demonstrating a 14% CRC mortality reduction with invitation to the program.^34^

This study also has limitations. Although we had access to individual level data on participants in SCREESCO, only aggregated data from the Stockholm–Gotland screening program were available and we did not have data on subsequent colonoscopies and findings. There may have been variations in Hb stability over time, but we did not have access to the batch numbers of the FITs. We also did not have data on other factors potentially associated with FIT outcomes, such as physical activity, gastrointestinal infections and inflammation, and transport and storage temperature of FITs. The number of false-negative FITs in SCREESCO was unknown since those individuals were not invited to colonoscopy. Our analysis of SCREESCO was performed in screening-naïve individuals and the performance of FIT may be different in individuals with a prior negative FIT and/or colonoscopy.

### Interpretation of findings

The impact of seasonal variations and temperature on FIT Hb concentrations and positivity has previously been reported in warmer regions, such as Italy, France, Germany, South Korea, and the United States.^5–15^ The relationship between seasonal variation in FIT Hb concentration and detection rate of CRC is however not clear. Some studies from Italy, the Netherlands, and the United States showed that seasonal variation in FIT concentration influenced the detection rate of advanced neoplasia or CRC,^5,7,9,14^ while others demonstrated no meaningful impact on detection rates.^6,8,10,12,13^ We found indications that during times when FIT Hb concentrations were higher and more individuals underwent colonoscopy, the risk of a false-positive FIT for detection of advanced neoplasia was higher when using a low cut-off (10 μg Hb/g feces). The seasonal variations were less distinct at higher cut-offs (40 and 80 μg Hb/g feces). This indicates that monitoring of fecal Hb concentration over time in screening is important to gain understanding of the variation in the performance and outcomes of screening, in particular when a lower cut-off is used. Such monitoring might enable more efficient use of colonoscopy resources by allowing adjustment of cut-off when positivity rates are unusually high or low.

The impact of sample return time of FIT has been widely examined in previous studies as a potential influence on FIT outcomes.^5,16–18^ Several studies suggest that delays in sample return, particularly under hot temperatures (e.g. 30 °C), might lead to a decrease in FIT positivity and colorectal neoplasia detection.^5,16–18^ However, in contrast to the cited studies, our research did not demonstrate a decrease in positivity with delayed sample return. This discrepancy is likely due to the cooler climate in northern Europe, although the median time between sampling and analysis was relatively short (3 days) in our study.

Our study elucidated several participant characteristics associated with FIT positivity. Higher age and male sex have been associated with increased FIT positivity also in other studies.^4–6^ Notably, a greater burden of comorbidities, as reflected by higher CCI and DCI in our study, was also linked to increased FIT positivity. This association merits attention in the context of individualization of screening strategies.^4^

## Conclusions

There was substantial variation in fecal Hb concentrations across calendar seasons of FIT screening in both the Swedish CRC screening trial SCREESCO and the regional CRC screening program of Stockholm–Gotland. The variation in the number of colonoscopies and detection rate of advanced neoplasia paralleled with this seasonal variation in FIT warrants further studies to optimize FIT-based CRC screening.

## Supplemental Material

sj-docx-1-msc-10.1177_09691413251369323 - Supplemental material for Variation in fecal hemoglobin concentrations: Cross-sectional analysis of a screening trial and a screening program in Sweden Supplemental material, sj-docx-1-msc-10.1177_09691413251369323 for Variation in fecal hemoglobin concentrations: Cross-sectional analysis of a screening trial and a screening program in Sweden by Masau Sekiguchi, Christian Löwbeer, Robert Steele, Johannes Blom, Anna Forsberg and Marcus Westerberg in Journal of Medical Screening

sj-pdf-2-msc-10.1177_09691413251369323 - Supplemental material for Variation in fecal hemoglobin concentrations: Cross-sectional analysis of a screening trial and a screening program in Sweden Supplemental material, sj-pdf-2-msc-10.1177_09691413251369323 for Variation in fecal hemoglobin concentrations: Cross-sectional analysis of a screening trial and a screening program in Sweden by Masau Sekiguchi, Christian Löwbeer, Robert Steele, Johannes Blom, Anna Forsberg and Marcus Westerberg in Journal of Medical Screening

sj-pdf-3-msc-10.1177_09691413251369323 - Supplemental material for Variation in fecal hemoglobin concentrations: Cross-sectional analysis of a screening trial and a screening program in Sweden Supplemental material, sj-pdf-3-msc-10.1177_09691413251369323 for Variation in fecal hemoglobin concentrations: Cross-sectional analysis of a screening trial and a screening program in Sweden by Masau Sekiguchi, Christian Löwbeer, Robert Steele, Johannes Blom, Anna Forsberg and Marcus Westerberg in Journal of Medical Screening
