# Supplementary material for: Variation in fecal hemoglobin concentrations: Cross-sectional analysis of a screening trial and a screening program in Sweden
Source: J Med Screen. 2025 Aug 21;33(1):29–37. doi: 10.1177/09691413251369323 (PMC12923636; doi:10.1177/09691413251369323)
Supplement: sj-docx-1-msc-10.1177_09691413251369323 - Supplemental material for Variation in fecal hemoglobin concentrations: Cross-sectional analysis of a screening trial and a screening program in Sweden [file sj-docx-1-msc-10.1177_09691413251369323.docx]

|  | **Cut-off 10 µg Hb/g** | | **Cut-off 40 µg Hb/g** | | **Cut-off 80 µg Hb/g** | |
| --- | --- | --- | --- | --- | --- | --- |
|  | **Odds ratio** | **95% confidence interval** | **Odds ratio** | **95% confidence interval** | **Odds ratio** | **95% confidence interval** |
| **Sex** |  |  |  |  |  |  |
| Male | 1.00 | Reference | 1.00 | Reference | 1.00 | Reference |
| Female | 0.75 | 0.71-0.81 | 0.70 | 0.63-0.78 | 0.69 | 0.61-0.80 |
| **Age at FIT (yearly increase)** | 1.02 | 0.96-1.10 | 1.04 | 0.96-1.15 | 1.07 | 0.98-1.23 |
| **Charlson comorbidity index** |  |  |  |  |  |  |
| 0 | 1.00 | Reference | 1.00 | Reference | 1.00 | Reference |
| 1 | 1.18 | 1.05-1.30 | 1.24 | 1.04-1.46 | 1.23 | 0.98-1.49 |
| 2 | 1.21 | 1.09-1.36 | 1.28 | 1.06-1.50 | 1.31 | 1.05-1.64 |
| ≥3 | 1.26 | 1.04-1.49 | 1.27 | 0.94-1.60 | 1.24 | 0.86-1.62 |
| **Drug comorbidity index**  (increase of size 1) | 1.22 | 1.19-1.26 | 1.2 | 1.15-1.25 | 1.25 | 1.19-1.32 |

**Supplementary Table 1.** Odds ratios from the multivariable models of categorical variables and continuous variables not represented using splines.
