# Supplementary material for: Variation in fecal hemoglobin concentrations: Cross-sectional analysis of a screening trial and a screening program in Sweden
Source: J Med Screen. 2025 Aug 21;33(1):29–37. doi: 10.1177/09691413251369323 (PMC12923636; doi:10.1177/09691413251369323)
Supplement: sj-pdf-2-msc-10.1177_09691413251369323 - Supplemental material for Variation in fecal hemoglobin concentrations: Cross-sectional analysis of a screening trial and a screening program in Sweden [file sj-pdf-2-msc-10.1177_09691413251369323.pdf]

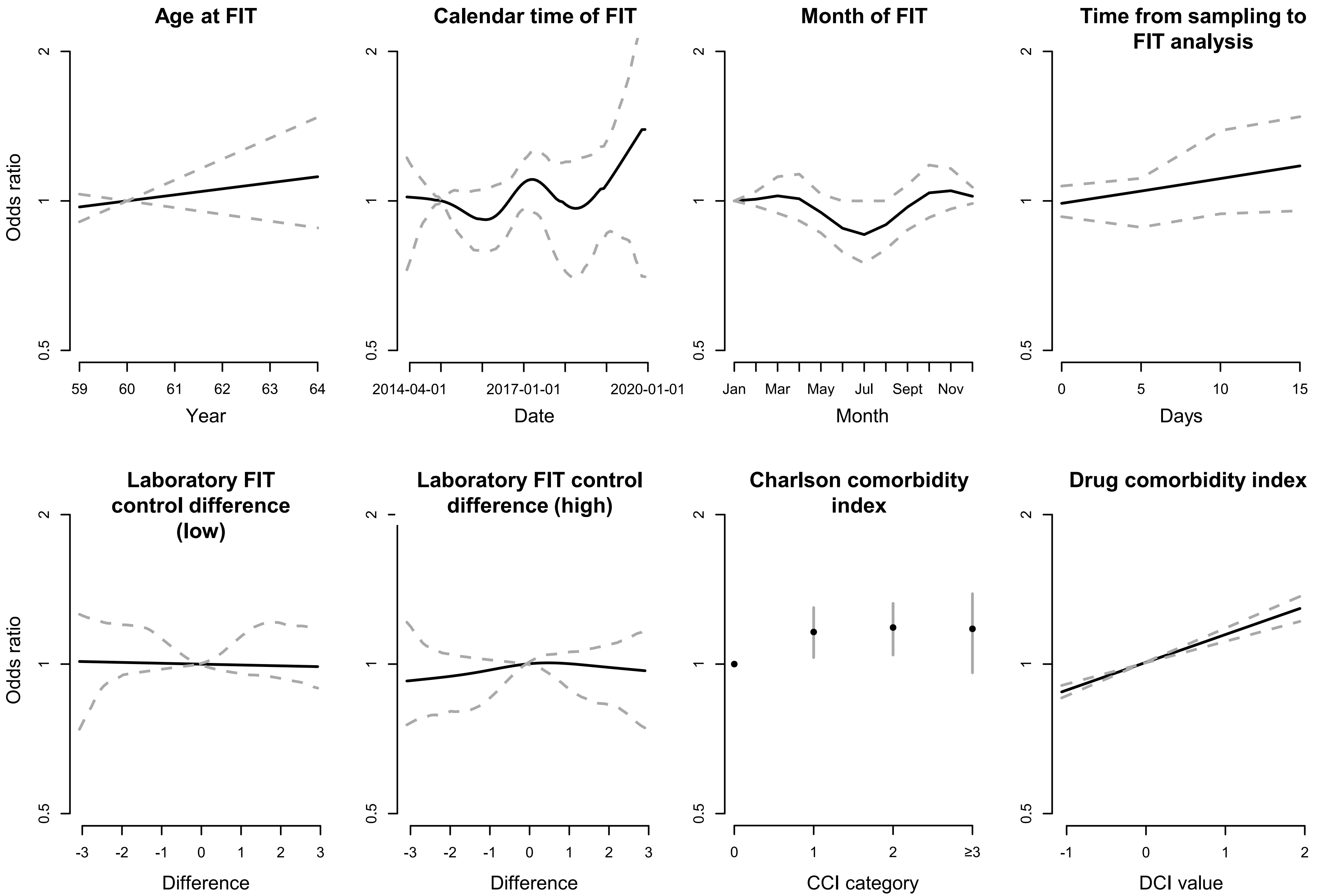

Supplementary figure 1. Odds ratios for FIT positivity (any of the two tests ≥ 40 µg Hb/g feces) in SCREESCO using a multivariable model. Dotted lines are for continuous variables and vertical bars (for Charlson comorbidity index) represent 95% confidence intervals.
